# Astragalus membranaceus–Derived Anti-Programmed Death-1 Monoclonal Antibodies with Immunomodulatory Therapeutic Effects against Tumors

**DOI:** 10.1155/2020/3415471

**Published:** 2020-03-03

**Authors:** Fu-Ling Chang, Keng-Chang Tsai, Tsai-Yu Lin, Tz-Wen Yang, Yan-Ni Lo, Wang-Chuan Chen, Jui-Hsien Chang, Mei-Kuang Lu, Chun-Tang Chiou, Po-Hung Chen, Yun Yen, Shiow-Lin Pan, Yu-Ching Lee

**Affiliations:** ^1^Ph.D. Program for Cancer Molecular Biology and Drug Discovery, College of Medical Science and Technology, Taipei Medical University and Academia Sinica, Taipei, Taiwan; ^2^National Research Institute of Chinese Medicine, Ministry of Health and Welfare, Taipei, Taiwan; ^3^The Ph.D. Program for Medical Biotechnology, College of Medical Science and Technology, Taipei Medical University, Taipei, Taiwan; ^4^TMU Research Center of Cancer Translational Medicine, Taipei Medical University, Taipei, Taiwan; ^5^The School of Chinese Medicine for Post Baccalaureate, I-Shou University, Kaohsiung, Taiwan; ^6^Department of Chinese Medicine, E-Da Hospital, Kaohsiung, Taiwan; ^7^Department of Health, Miaoli County Government, Miaoli, Taiwan; ^8^Department of Medical Technology, Jen-Teh Junior College of Medicine, Nursing and Management, Miaoli, Taiwan; ^9^Ph.D. Program for Cancer Molecular Biology and Drug Discovery, College of Medical Science and Technology, Taipei Medical University, Taipei, Taiwan; ^10^Ph.D. Program in Biotechnology Research and Development, College of Pharmacy, Taipei Medical University, Taipei, Taiwan; ^11^Biomedical Commercialization Center, Taipei Medical University, Taipei, Taiwan

## Abstract

*Astragalus membranaceus* polysaccharide (APS) components are main ingredients of TCM and have proven efficacy to activate T cells and B cells, enhancing immunity in humans. In this study, elevated cytokine and anti-PD-1 antibody titers were found in mice after immunization with APS. Therefore, phage-display technology was utilized to isolate specific anti-programmed death-1 (PD-1) antibodies from mice stimulated by APS and to confirm whether the isolated anti-PD-1 antibody could inhibit the interaction of PD-1 with the programmed death-ligand 1 (PD-L1), resulting in tumor growth inhibition. The isolated single-chain fragment variable (scFv) S12 exhibited the highest binding affinity of 20 nM to PD-1, completed the interaction between PD-1 and PD-L1, and blocked the effect of PD-L1-induced T cell exhaustion in peripheral blood mononuclear cells in vitro. In the animal model, the tumor growth inhibition effect after scFv S12 treatment was approximately 48%. However, meaningful synergistic effects were not observed when scFv S12 was used as a cotreatment with ixabepilone. Moreover, this treatment caused a reduction in the number of tumor-associated macrophages in the tumor tissue. These experimental results indirectly indicate the ability of APS to induce specific antibodies associated with the immune checkpoint system and the potential benefits for improving immunity in humans.

## 1. Introduction

*Astragalus membranaceus*, known as huang qi in Chinese, is a type of traditional Chinese medicine (TCM) commonly used as an immunomodulatory agent, and its extracts have been proven to effectively strengthen the immune system to treat pathological diseases and even cancer [1, 2]. The combined treatment strategy of administering *A. membranaceus* extract with other concurrent orthodox drugs (e.g., immunosuppressants and cancer chemotherapeutics) has also been shown to significantly reduce the toxicity of these drugs [3]. There are numerous critical biologically active components in the roots of dried *A. membranaceus*, such as polysaccharides, flavonoids, astragalosides, and saponins [4]. The composition of *Astragalus membranaceus* polysaccharide (APS) is highly complex, and numerous in vivo studies have confirmed its strong effect on immunomodulation. Administration of APS in experimental animals can promote the expression of cytokine interleukin- (IL-) 1*β*, tumor necrosis factor *α* (TNF-*α*), and interferon-*γ* (IFN-*γ*) genes and proteins [5, 6], which can effectively activate and increase the number of macrophages [7] and stimulate the proliferation of peripheral blood T cells [8]. Recently, one study showed that APS can enhance immunity by inducing the production of the cathelicidin-derived antimicrobial peptide LL-37 in human respiratory epithelial cells through the activation of the pathways of p38 MAPK/JNK and NF-*κ* [9]. Studies have demonstrated that in vaccine immunization, APS has great potential as a potent adjuvant, enhancing the effect of antigen presentation by improving the performance of class II major histocompatibility complex molecules, which facilitates lymphocyte proliferation, increases the number of antibodies in serum, and increases the secretion of cytokine [10, 11]. This echoes the immunomodulatory effects induced by APS to improve the state of humoral immunity by increasing the levels of immunoglobulin M (IgM) and immunoglobulin G (IgG) [12].

The discovery of immune checkpoints is a revolutionary breakthrough, and they have been applied in cancer treatment strategies, with the most representative being the interaction between programmed cell death protein 1 (PD-1) and programmed death ligand (PD-L1) [13]. PD-1 is expressed in activated T cells, B cells, natural killer cells, and bone marrow cells [14], whereas the PD-1 ligand PD-L1 is abundantly expressed in various tumors [15]. PD-L1 binds to the PD-1 on the surface of immune cells, inducing T cell failure and causing the inhibition of T cell activation and proliferation. Therefore, the interaction of PD-1 and its ligands, in particular PD-L1, facilitates immune evasion by cancer cells [13]. Numerous clinical trials on the targets of PD-1 or PD-L1 and combination therapy are currently underway [16]. TCMs' strong immunomodulatory functions for immune checkpoints have also been of great interest. Studies have demonstrated that APS can significantly inhibit the growth of melanoma cells in transgenic mice and reduce the expression of PD-L1 genes and proteins in tumors [17]. In addition, the Chinese medicine formula that contains astragalus (Bu-Fei Decoction) shows that the connection between tumor-associated macrophages (TAMs) and cancer cells can be disrupted by inhibiting the expression of IL-10 and PD-L1 molecules [18]. This suggests that the tumor-inhibiting mechanism of APS may be related to the regulation of PD-1–PD-L1 information pathways, which also enhance the antitumor immunocompetence of lymphocytes. However, because the composition of macromolecules such as polysaccharides is complex, the anticancer effects and mechanisms produced in vivo are still not fully understood. Therefore, whether such an immune response is related to the production of specific antibodies or correlated to the mechanism of immune checkpoint, blockade remains unclear and requires further investigation.

In this research, an alternative perspective was adopted to illustrate the possible effect of APS on the immunotherapy associated with immune checkpoints. The authors discovered that a specific anti-PD-1 antibody was induced in mice immunized with APS; thus, the immunomodulatory capacity of the isolated antibodies was investigated through indirect methods. Blocking the PD-1–PD-L1 interaction can maintain the activity of T cells and inhibit tumor growth. Although the reasons for the production of these induced antibodies are still unclear, the multifaceted ability of APS in immunomodulation is established and requires further investigation.

## 2. Materials and Methods

### 2.1. Animals

BALB/c mice were purchased from the National Laboratory Animal Center, Taiwan, and maintained in the animal facility of Taipei Medical University. The mice were maintained carefully, and all of the animal experiments followed ethical standards, with the protocols having been reviewed and approved by the Animal Use and Management Committee of Taipei Medical University (IACUC number: LAC-2013-0139).

### 2.2. APS Preparation and Mouse Immunization

The extract of APS dissolved in phosphate-buffered saline (PBS) was prepared for mouse immunization. APS was extracted from *A. membranaceus* following a previously described method [19]. For mouse immunization, female BALB/c mice 6 weeks of age were immunized with 50 *μ*g of APS with Freund's complete adjuvant (Sigma-Aldrich, Inc.) for the first time by intraperitoneal injection. Three additional immunizations were performed at intervals of 7 days, and the adjuvant was changed to Freund's incomplete adjuvant (Sigma-Aldrich, Inc.). The mouse spleen was harvested 7 days after the final immunization to construct an antibody library. The mouse sera were collected simultaneously to measure the levels of IL-2 and IFN-*γ* (Thermo Fisher Scientific Inc.) and the antibody titer against PD-1.

### 2.3. ScFv Library Construction and Biopanning

The mouse spleen was homogenized to isolate mRNA from spleen cells. After the reverse transcription of total mRNA into cDNA, mouse antibody genes (heavy chain and light chain variable regions) were amplified using specific antibody primers to construct a single-chain fragment variable (scFv) library through the application of phage-display technology. The method was according to the published protocols with minor modifications [20]. To enrich and isolate a specific clone from the constructed library through panning, recombinant phage particles (10^11^) from the constructed scFv antibody library were added to the well of a microtiter plate precoated with the recombinant PD-1 protein (1 *μ*g/well) and incubated at room temperature for 2 h. Unbound phages were subsequently removed, and the well was washed through pipetting with PBST (PBS with 0.05% Tween 20) 10 times. Bound phages were eluted with 0.1 M HCl-glycine (pH 2.2)/0.1% bovine serum albumin elution buffer and neutralized with 2 M Tris base buffer. The eluted phages were used to infect the *Escherichia coli* strain ER2738 immediately for recombinant phage amplification. Amplified phages were precipitated and recovered according to the described method [20] and used in the next round of panning. The panning procedure was repeated five times. After panning, total library DNA was purified and transformed into *E. coli* strain TOP 10F′ (Invitrogen, a nonsuppressor strain) for scFv expression. The expressed scFv was further purified with Ni^2+^-charged sepharose according to the manufacturer's instructions (GE Healthcare Life Sciences).

### 2.4. Sequence Analysis

To sequence the interested scFv clones, a primer ompseq (5′-AAGACAGCTATCGCGATTGCAGTG-3′) complementary to the outer membrane protein A (ompA) signal sequence in front of the light chain variable region was used. International ImMunoGeneTics information system/V-QUEry and Standardization (http://imgt.org) was used to compile and analyze the sequence data in accordance with the germline genes.

### 2.5. Enzyme-Linked Immunosorbent Assay

The wells of the microtiter plate were coated with recombinant human or mouse PD-1 protein (0.5 *μ*g/well) at 4°C overnight. After blocking with 5% skimmed milk, the purified scFv fused with the HA tag was added to the wells in duplicate and incubated for 1 h at room temperature. The scFv was washed with PBST, and the bound scFvs were then detected using mouse anti-HA tag antibodies (Proteintech Group, Inc.) and developed using horseradish peroxidase- (HRP-) conjugated goat anti-mouse IgG antibodies (Jackson ImmunoResearch Laboratories, Inc.). Finally, 3,5,5-tetramethubezidine (TMB) dihydrochloride substrate was added for signal development. The reaction was stopped by adding 1 N HCl, and absorbance was measured at 450 nm.

For phage enzyme-linked immunosorbent assay (ELISA), the amplified phage library obtained by each round of panning was added to the well that was coated with recombinant mouse PD-1 protein (0.5 *μ*g/well) and incubated. Bound phages were detected and developed using HRP-conjugated anti-M13 antibodies (GE Healthcare Lifescience).

### 2.6. Competitive Binding Assay

Competitive binding assays were used to determine the ability of scFv S12 to compete the interaction between PD-1 and PD-L1. In brief, wells of the microtiter plate were coated with recombinant PD-1 protein (0.5 *μ*g/well). After blocking with 5% skimmed milk for 1 h at room temperature, plates were washed with PBST twice. Then, various scFv S12 concentrations were added to the wells and were allowed to react with coated PD-1 protein. After incubation at room temperature for 1 h, plates were washed with PBST. Subsequently, human Fc-fused PD-L1 was added to each well and incubated for 1 h at room temperature. Goat antihuman Fc antibodies (Jackson ImmunoResearch Laboratories, Inc.) were added to detect bound PD-L1 molecules. Next, HRP-conjugated donkey anti-goat antibodies (Jackson ImmunoResearch Laboratories, Inc.) were used for the reaction. After washing, the TMB substrate was added to each well for development, and the reaction was stopped by adding 1 N HCl. The signal intensity was measured at OD 450 nm.

### 2.7. T Cell Activating Assay

T cell activation and proliferation were conducted using plate-immobilized anti-CD3 antibodies (eBioscience, Inc.) and PBMCs from healthy donors. Anti-CD3 monoclonal antibodies were coated on flat 96-well tissue culture plates at 4°C overnight. The next day, PBMCs were freshly prepared from peripheral blood using Ficoll-Paque (GE Healthcare Life Sciences) and labeled with 0.5 *μ*M CFSE (eBioscience, Inc.). Then, the CFSE-labeled PBMCs were incubated with testing scFv at room temperature for 30 min. The anti-CD3 antibody solution was removed, and the treated PBMCs were then distributed in the antibody-coated wells for incubation. T cell responses were analyzed 5 days after activation.

### 2.8. Tumor Growth Inhibition Assay

A mouse 4T1 breast tumor cell line was obtained from American Type Culture Collection (ATCC). Tumor cells used for implantation were harvested during the log phase of growth. BALB/c mice with the complete immune system were inoculated with these cells to form allograft tumors in the syngeneic animal model. When the tumors grew to approximately 200 mm^3^, the animals were divided into groups for the indicated scFv treatment. The scFv was administered by intravenous (i.v.) injection into the tail vein twice a week. Moreover, the small-molecule drug ixabepilone (Alvogen, Inc.) was administered by tail vein i.v. injection once weekly for combined treatment with scFv. The tumor size was measured twice weekly and calculated using *V* = 0.5 *lw*^2^, where *w* = width and *l* = length. For determining the tumor growth inhibition (TGI) effect, the antitumor effects were calculated by dividing the tumor volumes from the treatment groups by those from the control groups and multiplying by 100. The mice were examined frequently for overt signs of any adverse drug-related side effects.

### 2.9. Immunohistochemical Staining

The formalin-fixed and paraffin-embedded tumor tissues of the syngeneic animal model were prepared and sliced for immunohistochemical staining. The cell proliferation marker Ki-67, the macrophage surface markers CD68/CD163, and the T cell activating marker CD25 were stained with commercial antibody individually (Dako; Agilent Technologies, Inc. and Abcam, Inc.). Positive cells were calculated using the number of immunopositive cells × 100% divided by the total number of cells per field in 10 random fields at ×200 magnification. The results were captured using a Zeiss Axioskop-2 microscope.

## 3. Results

### 3.1. Evaluation of APS-Induced Immune Response

**Figure 1:**
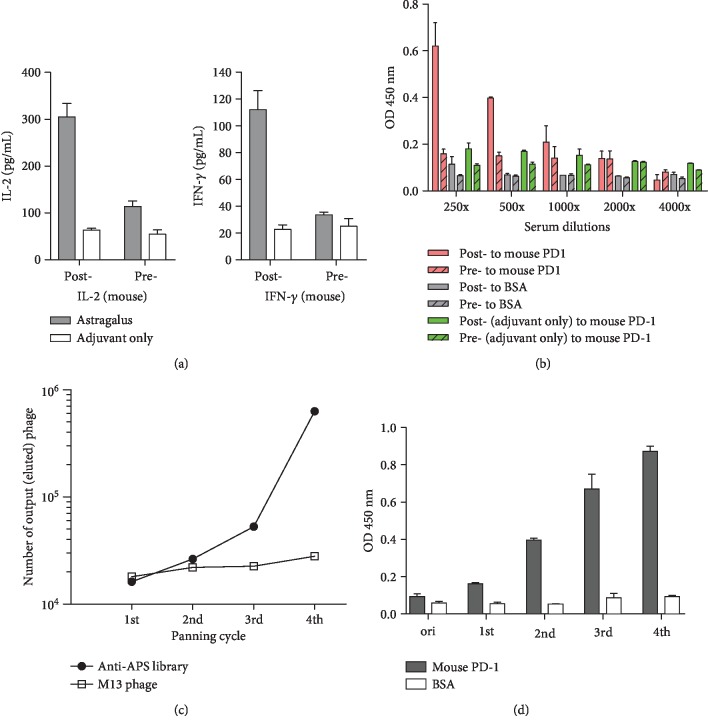
APS immune response and biopanning against PD-1. (a) Elevated IL-2 and IFN-*γ* levels in the mouse sera after immunization with APS were determined using ELISA. (b) The sera were tested using ELISA for the presenting titer of anti-PD-1 antibodies. Pre- and Post- denote whether the serum was collected before and after APS immunization. BSA is the negative antigen control. (c) The number of eluted phages binding to the mouse PD-1 protein after each round of panning of the anti-APS antibody library was calculated. Wild-type M13 phage was used as a negative library control in the panning. (d) The amplified antibody library phage after each round of panning was tested for binding to the mouse PD-1 protein using phage ELISA.

APS was extracted from *A. membranaceus* and used to immunize the mice and then evaluate the immune response. The experimental data showed that the levels of IL-2 and IFN-*γ* (Figure 1(a)) were significantly higher after immunization than before immunization, indicating increased T cell activity after immunization. To monitor the specific polyclonal antibody titer against PD-1 molecules after immunization, immune sera were 2-fold serially diluted (1 : 125 to 1 : 500), and their binding reactions with mouse PD-1 protein were tested. As shown in Figure 1(b), compared with the control group serum for adjuvant-only immunization, the serum for the fourth immunization of PD-1 exhibited a significantly higher identifiable anti-PD-1 antibody titer, which decreased as the dilution factor increased. To rule out that the antibody titer was caused by the increase in the total number of antibodies in the immunized mouse serum, the total quantity of mouse IgG in the diluted serum was measured, and no significant difference was detected (data not shown). This indicates that the binding reaction was not caused by the total IgG amount.

### 3.2. ScFv Antibody Library Construction and Biopanning

APS-immunized mice were sacrificed to construct a high-complexity (1.2 × 10^9^) phage-display scFv library and to conduct panning against mouse PD-1 protein. After each round of panning, the number of phages bound to the mouse PD-1 protein was calculated through elution, and the phage library was reamplified for the next round of panning. As shown in Figure 1(c), the number of eluted library phages increased with the round of panning. The number of bound phages in the fourth round was approximately 40-fold higher than that in the first round, which means that specific phage clones were enriched during the panning. However, the eluted phage number of control M13 wild-type phages in each round of panning was approximate. This data can be used as a key indicator of successful panning. In addition, after each round of panning, the recombinant phage library was tested using phage ELISA for determining its binding reactivity to mouse PD-1 protein, as shown in Figure 1(d). Compared with the original antibody library, the specific clones were enriched after the second to fourth rounds of panning and exhibited significant binding reactions.

### 3.3. Characterization of scFv Ability to Block the Interaction between PD-1 and PD-L1

**Figure 2:**
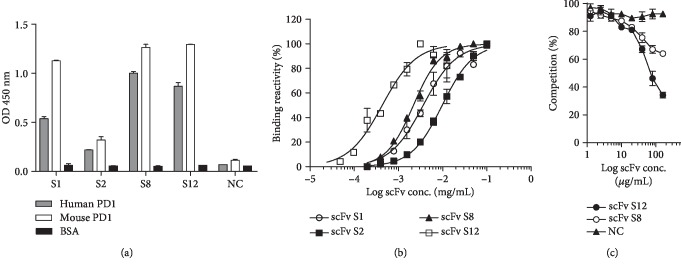
Binding reactivity of isolated scFv clones. (a) Binding reactivity of isolated scFvs to mouse or human PD-1 protein was determined through ELISA. (b) Binding reactivity of individual scFv of serially diluted concentration to the human PD-1 protein was determined. (c) The ability of scFvs to interfere with the interaction of human PD-1 and human PD-L1 proteins was determined using competitive ELISA. The quantity of bound Fc-fused PD-1 protein in the presence of free scFv as an inhibitor was measured and is expressed as a percentage of the binding of Fc-fused PD-1 in the absence of an inhibitor.

**Figure 3:**
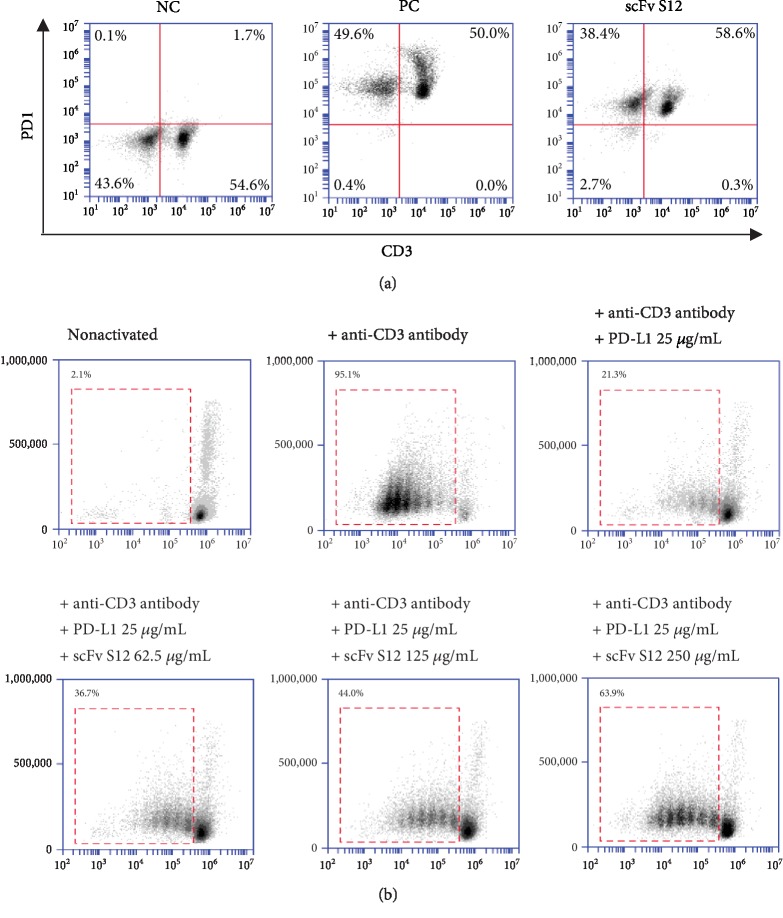
Testing the extent to which scFv S12 protected activated T cells in PBMCs by blocking the PD-1–PD-L1 interaction. (a) The corresponding antibodies were used to detect the expression of CD3^+^ and PD-1^+^ cells in the freshly prepared PBMC and to test the effect of scFv S12 in identifying endogenous human PD-1 in PBMC. NC represents the results of staining within anti-CD3 antibodies, PC is the result of double staining with anti-PD-1 and anti-CD3 antibodies, and scFv S12 is the result of double staining using scFv S12 and anti-CD3 antibodies. (b) Various concentrations of scFv S12 were tested to establish how much protection they provided to activated T cells in PBMC through the blocking of the PD-1–PD-L1 interaction. Anti-CD3 antibody could effectively activate T cells to induce a proliferative response. However, such proliferation and differentiation could be inhibited in the presence of PD-L1 molecules. The results of cell proliferation were determined through CSFE cell staining.

After panning, four representative clones were isolated for single-colony analysis. We used the mouse PD-1 protein for panning. However, mouse and human PD-1 proteins are homologous, and these mouse-derived scFvs tested their reactivity of cross-binding to human PD-1. As seen in Figure 2(a), four isolated scFvs all had binding ability to both mouse and human PD-1 proteins. Based on the serially diluted scFv concentration, we determined the binding reactivity of the four isolated scFvs to the human PD-1 protein. scFv S12 had the best binding affinity; EC_50_ was calculated to be 20 nM approximately (Figure 2(b)). Moreover, the most effective two scFvs, scFv S8 and S12, were confirmed to have the ability to block the interaction of PD-1 and PD-L1. The results showed that scFv S12 had better affinity and significantly interfered with the interaction to 70% (Figure 2(c)). Furthermore, we tested the ability of scFv S12 for the inhibition of T cell exhaustion in freshly prepared peripheral blood mononuclear cells (PBMCs). In Figure 3(a), the binding reactivity of scFv S12 to the endogenous PD-1 on the surface of T cells was the first to be confirmed (CD3^+^, PD-1^+^) in PBMC. As seen in Figure 3(b), in the control group without scFv treatment, PD-L1 of 25 g/mL could inhibit the proliferation of activated T cells from 95% to 21.3%. The results indicate that scFv S12 protects the proliferative response of T cell activation and demonstrates a dose-dependent effect.

### 3.4. Inhibitory Effect of Anti-PD-1 scFv S12 on 4T1 Mouse Allograft Model

**Figure 4:**
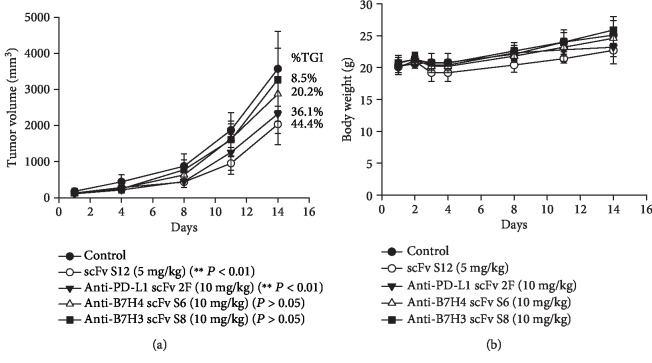
The tumor growth inhibitory effect of anti-PD-1 scFv S12 on the 4T1 mouse allograft model was tested. BALB/c mice with 4T1 allograft tumors established subcutaneously were randomly assigned to five groups (*n* = 5 per group) and received the indicated treatment. Other self-developed scFv antibodies for the immune checkpoints were added for comparative testing. (a) For the scFv S12 group, 5 mg/kg of the antibody was administered through intravenous injection twice a week; other scFv groups were administered 10 mg/kg of the antibodies through intravenous injection twice a week. The tumor growth curves are presented as the mean ± SD, and TGI (%TGI) was also calculated. ^∗∗^The difference at the level of *P* < 0.01 was obtained through comparison with the untreated control group. (b) The body weights of the mice were measured on the first 4 days of the first week after administration of the antibodies after which their weight was measured every 3 days until the conclusion of the experiment. The body weight is presented as the mean ± SD.

**Figure 5:**
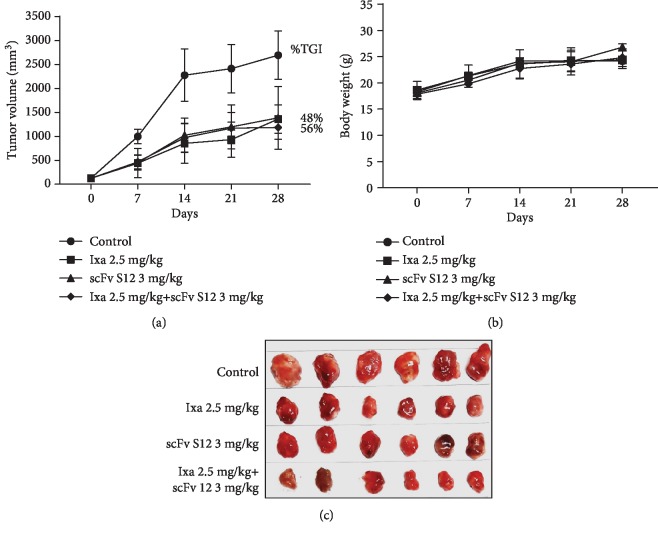
The tumor inhibitory effect of anti-PD-1 scFv S12 combined with the small-molecule drug ixabepilone for combination therapy was tested. BALB/c mice with 4T1 allograft tumors established subcutaneously were randomly assigned to four groups (*n* = 6 per group) and received the designated treatment. (a) Ixa (ixabepilone) was administered by intravenous injection once a week at a dose of 2.5 mg/kg, whereas scFv S12 was administered via intravenous injection twice a week at a dose of 3 mg/kg. In addition, a group underwent combination therapy with Ixa and scFv S12. The tumor growth curves are presented as the mean ± SD, and the tumor growth inhibition (%TGI) was also calculated. ^∗∗^The difference at the level of *P* < 0.01 was obtained through comparison with the untreated control group. (b) The body weight of the mice was measured once every 7 days, after the antibody was administered, until the experiment concluded. Their body weights are presented as the mean ± SD. (c) Tumors of individual mice were extracted at the end of the experiment for observation of the tumor size.

The TGI effect of scFv S12 was determined in the 4T1 mouse allograft model treatment.  Figure 4(a) shows that after i.v. injection with a 5 mg/kg dosage of scFv twice weekly, scFv S12 could inhibit tumor growth at a tumor growth inhibition rate (%TGI) of 44.4%. Compared with the effects observed for other self-developed immune checkpoint inhibitors (scFvs) administered at 10 mg/kg dosages twice weekly (including anti-PD-L1, anti-B7H3, and anti-B7H4, the results for which have not been published), the inhibitory effect of scFv S12 was found to be superior. During the treatment, scFv S12 had no toxicity and did not affect the animals' body weight (Figure 4(b)). Moreover, in another allograft animal experiment, the possible synergistic effects of scFv S12 combined with small-molecule drugs were tested. As seen in Figure 5(a), i.v. injection with scFv S12 at a 3 mg/kg dosage twice weekly or i.v. injection with ixabepilone at a 2.5 mg/kg dosage once per week separately demonstrated similar inhibitory effects at the %TGI of approximately 48% (Figure 5(a)). The scFv treatments had no toxicity and did not affect animals' body weight (Figure 5(b)). Although the inhibitory effect of scFv S12 combined with ixabepilone was 56%, the difference was nonsignificant. Observation of the dissected tumor tissue revealed that after scFv S12 treatment, the growth of tumors was suppressed (Figure 5(c)). Notably, the effect of the combined treatment seemed to be slightly enhanced.

### 3.5. Immune System Alteration in Tumor Microenvironment after scFv S12 Treatment

**Figure 6:**
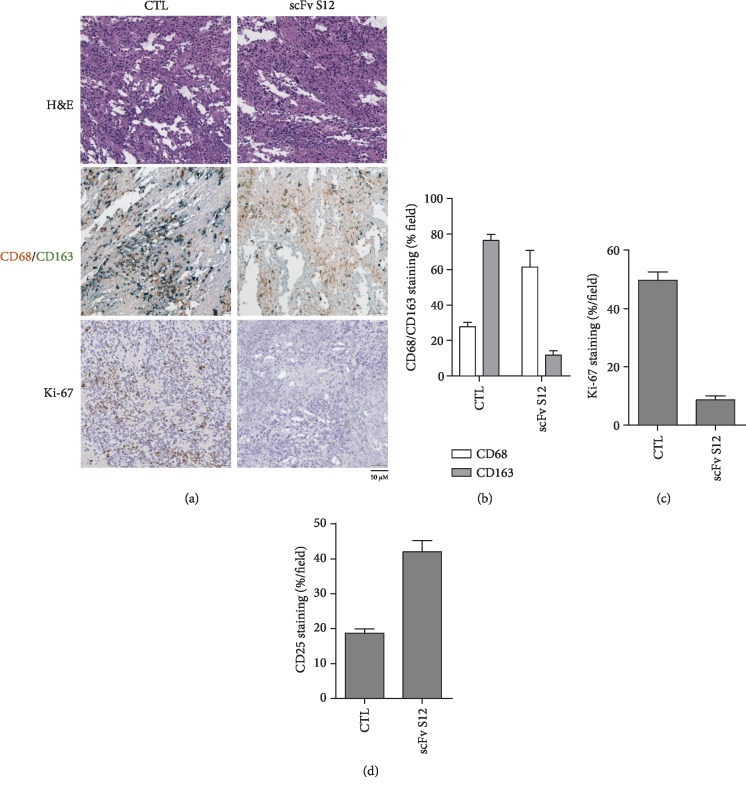
Changes in the intrinsic molecules of the mouse tumor after treatment were observed through immunohistochemical staining. (a) The anti-CD68/CD163 antibody was used to double-stain tumor tissue to observe the distribution and infiltration of M1 and M2 phase macrophages in tissues, and the expression of the cell proliferation marker Ki-67 was observed through the anti-Ki-67 antibody. (b) The CD68/CD163 ratio presented in the scFv S12 treatment group and the untreated control group in 10 random fields was calculated and quantified. (c) The staining results for Ki-67 presented in the scFv S12 treatment group and the untreated control group in 10 random fields were calculated and quantified. (d) The staining results for T cell activating marker CD25 presented in the scFv S12 treatment group and the untreated control group in 10 random fields were calculated and quantified.

The tumor tissue was immunohistochemically stained to examine the effect of an anti-PD-1 inhibitor on TAM distribution. As seen in Figures 6(a) and 6(b), the level of M1-phenotype macrophages (with surface marker staining for CD68) and M2-phenotype macrophages (with surface marker staining for CD163) demonstrated a reverse change. The antitumor activity in the immune system increased because of inflammation and immunostimulation in the tumor microenvironment. As compared with the control group, which received no treatment, the staining of the cell proliferation marker Ki-67 significantly decreased in the tumor tissues after scFv S12 treatment, implying that the tumor growth was inhibited by immunomodulatory effects after scFv treatment (Figure 6(c)). Moreover, the staining of T cell activating marker CD25 that increased in the tumor tissues after scFv S12 treatment is another evidence to support the effect of immune checkpoint blockade usage (Figure 6(d)).

## 4. Discussion

The therapeutic strategies for enhancing immunity using *A. membranaceus* and based on TCM theories have been confirmed clinically and theoretically. After APS was used to immunize the mice in the current experiment, the potency of the anti-PD-1 antibody measured in the serum (Figure 1(b)) was less significant than that for animals directly immunized with the protein. However, the experimental data exhibited a difference, indicating that the anti-PD-1 antibody in the serum had been specifically induced. Instead of rapid effects, the TCM treatment strategy focuses on sustained effects, which are usually the result of the long-term oral administration and continuous use of TCMs. This highlights the question of whether, after using the *A. membranaceus* extract, the indicator component APS can induce a low dose of anti-PD-1 antibody response to protect activated T-lymphocyte activity and sustain a low-concentration antibody potency. To understand whether the antibodies induced in the body possess actual functionality, the authors isolated the antibodies with specific binding ability to PD-1 proteins through indirect methods using phage-display technology. In addition to specifically binding to PD-1, these isolated antibodies effectively blocked the interaction between PD-1 and PD-L1 (Figures 2(b) and 2(c)). Moreover, in in vitro experiments, the antibodies effectively blocked the weakened reaction of activated T cells in PBMC affected by PD-L1. Therefore, these results also indicate that the anti-PD-1 antibody response elected after immunization with APS in vivo possesses immunomodulatory function.

In animal experiments, the mouse tumor 4T1 allograft model was used to conduct TGI testing. Other antibody drugs that were also aimed at the immune checkpoint were added in the experiment, and their ability to recognize mouse proteins was confirmed before use (data not shown). The results showed that the anti-PD-1 antibody (scFv S12) was the most effective, followed by the anti-PD-L1 antibody (scFv 2F). Because a syngeneic animal model was used, the tumor growth was relatively rapid, and the growth inhibition effect for the tumor was potentially caused by the activated immune cells. The effect of TGI using anti-PD-1 antibodies demonstrated similar TGI responses (44.4% and 48.6%) in the two animal experiments (Figures 4(a) and 5(a)). Because the approach of targeting PD-1 and PD-L1 immune checkpoint inhibitors in cancer treatment has been successful, various clinical experiments are being conducted on the use of self-immunomodulatory characteristics for combining immune checkpoint inhibitors and small-molecule drugs for cancer treatment strategies [16]. Ixabepilone is an FDA-approved small-molecule drug for the treatment of metastatic or locally advanced breast cancer [21], and the drug was found to exert a synergistic effect with the blocking effect of CTLA-4 [22]. In the current study, ixabepilone was combined with anti-PD-1 scFv S2, and their synergistic effect was tested. Although the final result displayed a 7% difference in tumor size calculation, and slight changes were observed in the tumors extracted after treatment, the synergistic effect was nonsignificant (*P* > 0.05). Although the APS-induced anti-PD-1 antibody did not show a superior TGI effect as compared to the drug group in the study (Figure 5(a)), the results suggested the possibility of using APS to enhance immunomodulation and to be a complement of the treatment. The use of APS may allow sustaining an effective dose of anti-PD-1 antibody in the body, and it may delay the progression of tumor or tumorigenesis by increasing the activity of T cells. In cancer therapy, there is still an opportunity to combine the APS treatment with small-molecule drugs to improve or induce the synergistic effect.

Molecular changes in the tumor microenvironment substantially affect tumor growth. TAMs exhibit notable correlations in tumor progression, metastasis, and posttreatment recurrence, and the macrophages are classified into M1 and M2 based on their plasticity and diversity [23]. The main role of M1 macrophages is to kill pathogens and tumor cells, whereas M2 macrophages promote tumor development. The polarization states and distribution of M1 and M2 macrophages have been shown to influence the course of cancer and responses to treatment [24]. M2 macrophages directly facilitate angiogenesis and tumor matrix remodeling and invasion through the secretion of various growth factors and proteolytic enzymes [25–27]. In addition, M2 macrophages can directly facilitate tumor development by inhibiting antitumor immune responses by secreting immunosuppressive factors such as IL-10 and transforming growth factor beta (TGF-*β*) and by attracting regulatory T cells to tumor tissues to inhibit the proliferation and activation of T cells [28]. One study indicated that administering anti-PD-1 antibodies altered the composition of the tumor microenvironment, which is attributable to the activation of M1 macrophages and the reduction of M2 macrophages in tumors [29]. The same response was observed in the treated tumor tissue in the present study. As seen in Figure 6(a), after treatment with PD-1 antibodies, the ratio of M1 and M2 macrophages in the tumor tissue displayed significant changes compared with the ratio in the control group. The decline in M2 macrophages indicated that the immune response redirected the polarization state of macrophages, whereas the increase in M1 macrophages suggested an increase in antitumor effects through in vivo immunomodulation. This also indicated that the tumor microenvironment must have contained sufficient effective immune cells to reverse the situation, producing a reaction inhibiting tumor growth.

## 5. Conclusions

Finally, based on the results of the experiment, the authors confirm that APS, the active component in *A. membranaceus*, can induce low doses of anti-PD-1 antibody responses in animals, and these antibodies may have immunomodulatory functions that can overcome the failure of immune cells under immune evasion by tumors. These results are an attempt to illustrate the possible role of *A. membranaceus* in immunomodulation from an alternative perspective and can assist in providing more research information for the development of anticancer drugs.

## Data Availability

No data were used to support this study.

## References

[1] Auyeung K. K., Han Q. B., Ko J. K. (2016). Astragalus membranaceus: a review of its protection against inflammation and gastrointestinal cancers. *The American Journal of Chinese Medicine*.

[2] Zhao L. H., Ma Z. X., Zhu J., Yu X. H., Weng D. P. (2011). Characterization of polysaccharide from Astragalus radix as the macrophage stimulator. *Cellular Immunology*.

[3] Wang S. F., Wang Q., Jiao L. J. (2016). Astragalus-containing traditional Chinese medicine, with and without prescription based on syndrome differentiation, combined with chemotherapy for advanced non-small-cell lung cancer: a systemic review and meta-analysis. *Current Oncology*.

[4] Zhao M., Zhang Z. F., Ding Y., Wang J. B., Li Y. (2012). Astragalus polysaccharide improves palmitate-induced insulin resistance by inhibiting PTP1B and NF-*κ*B in C2C12 myotubes. *Molecules*.

[5] Li J., Bao Y., Lam W. (2008). Immunoregulatory and anti-tumor effects of polysaccharopeptide and Astragalus polysaccharides on tumor-bearing mice. *Immunopharmacology and Immunotoxicology*.

[6] Yuan C., Pan X., Gong Y. (2008). Effects of Astragalus polysaccharides (APS) on the expression of immune response genes in head kidney, gill and spleen of the common carp, Cyprinus carpio L. *International Immunopharmacology*.

[7] Wang J., Ito H., Shimura K. (1989). Enhancing effect of antitumor polysaccharide from Astragalus or Radix hedysarum on C3 cleavage production of macrophages in mice. *Japanese Journal of Pharmacology*.

[8] Li S. P., Zhao X. J., Wang J. Y. (2009). Synergy of Astragalus polysaccharides and probiotics (Lactobacillus and Bacillus cereus) on immunity and intestinal microbiota in chicks. *Poultry Science*.

[9] Zhao L., Tan S., Zhang H. (2018). Astragalus polysaccharides exerts anti-infective activity by inducing human cathelicidin antimicrobial peptide LL-37 in respiratory epithelial cells. *Phytotherapy Research*.

[10] Chen Y., Wang D., Hu Y. (2010). Astragalus polysaccharide and oxymatrine can synergistically improve the immune efficacy of Newcastle disease vaccine in chicken. *International Journal of Biological Macromolecules*.

[11] Yang F., Xiao C., Qu J., Wang G. (2016). Structural characterization of low molecular weight polysaccharide from Astragalus membranaceus and its immunologic enhancement in recombinant protein vaccine against systemic candidiasis. *Carbohydrate Polymers*.

[12] Guo L., Liu J., Hu Y. (2012). Astragalus polysaccharide and sulfated epimedium polysaccharide synergistically resist the immunosuppression. *Carbohydrate Polymers*.

[13] Wu X., Gu Z., Chen Y. (2019). Application of PD-1 blockade in cancer immunotherapy. *Computational and Structural Biotechnology Journal*.

[14] Kedmi M., Avigdor A., Nagler A. (2015). Anti-PD-1-targeted therapies focusing on lymphatic malignancies: biological rationale, clinical challenges and opportunities. *Acta Haematologica*.

[15] Patel S. P., Kurzrock R. (2015). PD-L1 expression as a predictive biomarker in cancer immunotherapy. *Molecular Cancer Therapeutics*.

[16] Burugu S., Dancsok A. R., Nielsen T. O. (2018). Emerging targets in cancer immunotherapy. *Seminars in Cancer Biology*.

[17] Wang J. R., Wang J. Y., Zhang T. T., Cheng X. D. (2014). Regulate molecular expression of PD-1/PD-Ls by Astragalus polysaccharides on melanoma mice. *Acta Universitatis Traditionis Medicalis Sinensis Pharmacologiaeque Shanghai*.

[18] Pang L., Han S., Jiao Y., Jiang S., He X., Li P. (2017). Bu Fei decoction attenuates the tumor associated macrophage stimulated proliferation, migration, invasion and immunosuppression of non-small cell lung cancer, partially via IL-10 and PD-L1 regulation. *International Journal of Oncology*.

[19] Huang Y. C., Tsay H. J., Lu M. K. (2017). Astragalus membranaceus-polysaccharides ameliorates obesity, hepatic steatosis, neuroinflammation and cognition impairment without affecting amyloid deposition in metabolically stressed APPswe/PS1dE9 mice. *International Journal of Molecular Sciences*.

[20] Barbas C. F., Burton D. R., Scott J. K., Silverman G. J. (2001). *Phage Display A Laboratory Manual*.

[21] Lopus M., Smiyun G., Miller H., Oroudjev E., Wilson L., Jordan M. A. (2015). Mechanism of action of ixabepilone and its interactions with the *β*III-tubulin isotype. *Cancer Chemotherapy and Pharmacology*.

[22] Jure-Kunkel M., Masters G., Girit E. (2013). Synergy between chemotherapeutic agents and CTLA-4 blockade in preclinical tumor models. *Cancer Immunology, Immunotherapy*.

[23] Genard G., Lucas S., Michiels C. (2017). Reprogramming of tumor-associated macrophages with anticancer therapies: radiotherapy versus chemo- and immunotherapies. *Frontiers in Immunology*.

[24] Cao L., Che X., Qiu X. (2019). M2 macrophage infiltration into tumor islets leads to poor prognosis in non-small-cell lung cancer. *Cancer Management and Research*.

[25] Du R., Lu K. V., Petritsch C. (2008). HIF1*α* Induces the Recruitment of Bone Marrow-Derived Vascular Modulatory Cells to Regulate Tumor Angiogenesis and Invasion. *Cancer Cell*.

[26] Gocheva V., Wang H. W., Gadea B. B. (2010). IL-4 induces cathepsin protease activity in tumor-associated macrophages to promote cancer growth and invasion. *Genes & Development*.

[27] Gil-Bernabé A. M., Ferjančič Š., Tlalka M. (2012). Recruitment of monocytes/macrophages by tissue factor-mediated coagulation is essential for metastatic cell survival and premetastatic niche establishment in mice. *Blood*.

[28] Kryczek I., Zou L., Rodriguez P. (2006). B7-H4 expression identifies a novel suppressive macrophage population in human ovarian carcinoma. *The Journal of Experimental Medicine*.

[29] Dhupkar P., Gordon N., Stewart J., Kleinerman E. S. (2018). Anti-PD-1 therapy redirects macrophages from an M2 to an M1 phenotype inducing regression of OS lung metastases. *Cancer Medicine*.

